# Untargeted Metabolomics for Diagnosis, Monitoring, and Understanding the Pathophysiology of Inherited Metabolic Disorders

**DOI:** 10.1002/jimd.70120

**Published:** 2025-12-02

**Authors:** Jonathan Martens, Udo F. H. Engelke, Ron A. Wevers, Dirk J. Lefeber, Purva Kulkarni

**Affiliations:** ^1^ Institute for Molecules and Materials, FELIX Laboratory Radboud University Nijmegen the Netherlands; ^2^ Department of Human Genetics Translational Metabolic Laboratory, Radboud University Medical Centre Nijmegen the Netherlands; ^3^ United for Metabolic Diseases (UMD) Nijmegen the Netherlands; ^4^ Department of Neurology Donders Institute for Brain, Cognition and Behavior, Radboud University Medical Center Nijmegen the Netherlands

**Keywords:** biomarkers, dark matter of the metabolome, de‐VUSing, diagnostics, inborn errors of metabolism, mass spectrometry, metabolic disease discovery, untargeted metabolomics

## Abstract

Inherited metabolic disorders (IMDs) encompass a diverse and expanding group of rare diseases caused by genetic disruptions mainly in metabolic enzymes and transporters. Clinical diagnosis of IMDs presents significant challenges due to phenotypic heterogeneity, nonspecific symptoms, and the limited scope of current targeted biochemical assays typically available. Recent advances in mass spectrometry‐based untargeted metabolomics offer promising solutions to several of these challenges by simultaneous detection and relative quantification of thousands of metabolites, not relying on any prior hypotheses. With the expansion of genetic diagnostics via whole‐exome and whole‐genome sequencing, metabolic insights are often crucial for understanding the pathogenicity of genetic variants of unknown significance, often enabling a clear diagnosis for patients. This review details current applications of untargeted metabolomics in IMDs, including biomarker discovery and elucidation of previously unknown pathophysiological mechanisms. Successful examples of biomarker identification in well‐studied IMDs, such as pyridoxine‐dependent epilepsy and phenylketonuria, are highlighted to provide novel disease insights. Additionally, we address technical and interpretation challenges inherent to this methodology, particularly concerning metabolite identification, high‐dimensional data complexity, and limited patient numbers. Emerging analytical technologies and data analysis approaches are highlighted that are poised to mitigate these challenges in the upcoming years. Finally, we provide an outlook on future directions, emphasizing the complementary roles of targeted and untargeted metabolomics and the prospects for the identification of new therapeutic targets as well as therapy monitoring for the clinical management of IMDs.

## 1 Introduction

Inherited metabolic disorders (IMDs) are a large, heterogeneous group of disorders typically caused by mutations in genes encoding metabolic enzymes or metabolite transporters. Although each IMD is individually rare, these disorders are collectively prevalent affecting ~1 in 500 births [1]. The international classification of inherited metabolic disorders (ICIMD), [2] provides a comprehensive, hierarchical, group‐based collation of over 1460 known IMDs. As of January 2025, the Inborn Errors of Metabolism Knowledgebase (IEMbase, http://www.iembase.org) [3], an online expert‐curated resource for IMDs, reported a total of over 1900 such disorders.

Diagnosis is important, since IMDs can significantly contribute to morbidity, diminished well‐being, and even mortality, especially in the early years of life. The diagnosis of IMDs can be very challenging. The clinical and phenotypic presentation of these conditions spans a wide spectrum, ranging from the involvement of a single organ to multiple different organs. Symptoms may be acute, as sometimes seen in childhood urea cycle disorders, progressive, as commonly observed in lysosomal storage disorders, or more stable over life as in McArdle's disease. In addition, patients with the same metabolic disorder can present with widely different phenotypic severities, such as neonatal hydrops and severe multisystem disease in PMM2‐CDG neonates versus mild ataxia in adult patients with the same disease [4, 5]. Moreover, completely different clinical entities have been linked to the same gene defect, for example, vacuolar myopathy [6] or vacuolar hepatopathy [7] due to *VMA21* mutations, depending on the type of mutation. It is evident from research conducted over the years that a growing number of these conditions are treatable. This makes early diagnosis crucial to initiate disease management and timely therapy to prevent severe complications. An example of a well‐known IMD is phenylketonuria (PKU, OMIM 261600) [8, 9]. PKU is an autosomal recessive IMD, where the body cannot properly process phenylalanine due to a deficiency of the phenylalanine hydroxylase enzyme. Without prompt treatment, toxic levels of phenylketones will accumulate, eventually leading to severe and irreversible intellectual disabilities, epilepsy, and behavioral disorders. By diagnosing PKU early, dietary management and specialized treatments can be initiated, enabling the affected individual to lead a healthy life [10].

Several IMDs with known biomarkers are now routinely screened for at birth as a part of newborn screening programs, which is usually the first‐tier assessment of IMDs [11, 12, 13]. In most developed countries and several low‐income countries, each newborn is screened for IMDs by dried blood spot testing [14] and analyzed using direct infusion mass spectrometry (MS). However, due to the molecular and clinical variability of IMDs, most still need to be diagnosed by selective screening on specific requests depending on the clinical suspicion of the treating physician and their clinical experience. This standard biochemical testing is often done sequentially and may need repeated sampling. This can be time‐consuming and may cause diagnostic delay, which, given the various highly specific therapies, is undesirable. Additionally, several IMDs also lack reliable biomarkers to develop such specific biochemical assays. A gene mutation in an IMD disrupts a metabolic pathway, causing a metabolic roadblock, resulting in an excessive accumulation or deficit of certain essential biomolecules within the body. The diagnosis of IMDs by uncovering metabolite perturbations has long been performed by applying a combination of routine first‐tier biochemical laboratory tests on patient body fluids (urea and electrolytes, liver function tests, blood gases, blood glucose concentrations, plasma ammonia, etc.). These are also sometimes performed together with second‐line targeted metabolic investigations (plasma amino acids, acylcarnitine profile, urinary organic acids, and urinary glycosaminoglycans (=GAGs), etc.) before proceeding to specific metabolic tests (e.g., very long chain fatty acids or lysosomal enzymes) [15, 16]. Targeted approaches, typically using MS, enable absolute quantification for a specific subset of known metabolites. However, these targeted approaches rely more heavily on clinical symptomatology typically guiding the selection of specific analyses for each patient. This may lead to a risk of false negatives if metabolic tests are missed due to nonspecific symptoms. Additionally, novel metabolic perturbations are often completely overlooked.

With advancements in next‐generation sequencing technologies over the last decades, the use of clinical exome panels or whole‐exome sequencing (WES) and whole‐genome sequencing (WGS) has significantly increased the diagnostic rate of IMDs [17, 18, 19, 20]. Whole‐exome sequencing involves sequencing all protein‐coding regions of the genome, referred to as the exome, to identify pathogenic mutations. It is extremely powerful at detecting a Mendelian disease gene in a single patient [21]. WES in inconclusive cases has also helped to identify new genes and novel IMDs [22, 23]. It has become commonplace in the diagnostic arena and is being applied especially in cases of unknown etiology. This is useful in IMDs where many genes can cause similar disease phenotypes such as mitochondrial disorders [24, 25] but also aids in finding the molecular cause when a phenotype is atypical for a known disease gene [26], or when a disease gene is associated with an unexpected metabolic pathway [27]. Many of the new IMD genes, however, encode sparsely characterized proteins, which offer limited disease insight and no treatment options. At the same time, genetic testing has identified many IMD cases where the clinical significance of the defect is uncertain, due to challenges in variant prioritization as well as interpreting and identifying variants of uncertain significance (VUS) [28]. This limitation has been increasingly addressed by applying metabolomics as a functional screen [29, 30, 31]. Metabolomics is the measurement and study of a vast array of small molecules called metabolites, typically < 1000 Da, in biological samples (e.g., tissues, body fluids, and cells), constituting the metabolome of an organism. Metabolomics is widely recognized as the omics discipline most closely linked to the phenotype [32]. These functional products are of course influenced by gender, age, diet, the environment, lifestyle, the microbiome, and drugs and also by disease, and particularly IMDs. The measured metabolites, hence, can act as markers for a variety of purposes: diagnostic biomarkers (to detect or confirm the presence of a disease), prognostic biomarkers (that can predict the likely course or outcome of a disease), monitoring biomarkers (to assess the effect of therapy and disease progression), and predictive biomarkers (that can provide information on the probability of a response or the lack of it to a particular therapy), among others.

More recently, untargeted MS‐based approaches have been implemented in clinical diagnostics to characterize a wide range of metabolites rather than a specific subset [33]. These approaches, collectively referred to as untargeted metabolomics, serve as an unbiased tool to identify and study both known and novel biochemical perturbations in the metabolome of the measured sample. In this review, we first focus on the application of untargeted metabolomics in a clinical laboratory setting for the diagnosis of IMDs. In the later sections, we provide several examples of using untargeted metabolomics for biomarker discovery and shed light on the associated challenges to fully exploit untargeted metabolomics in the future.

## 2 Application of Untargeted Metabolomics for the Diagnosis of IMDs

While targeted assays have long been the standard in metabolic diagnostic laboratories, offering reliable results with precise quantification, there has been a growing need for a more comprehensive test to provide a complete overview of the metabolic status. This need arose due to several factors: (1) conducting multiple targeted assays on a single sample was time‐, money‐, and labor‐intensive, resulting in longer turnaround times, and (2) as new biomarkers and IMDs were continuously being reported, a more comprehensive test became essential [34]. The application of untargeted metabolomics fills this gap and enables powerful, unbiased relative quantification of thousands of endogenous metabolites in a single measurement.

A typical untargeted metabolomics workflow can be broadly divided into five main components: (1) sample preparation, (2) sample measurement and detection of metabolites, (3) data preprocessing, (4) metabolite annotation, and (5) data analysis and interpretation.

### 2.1 Sample Preparation

The samples or body fluids typically measured to perform metabolite profiling in a metabolic diagnostic laboratory include blood plasma, dried blood spots, urine, or cerebrospinal fluid samples, the choice of which depends on the patient's clinical phenotype. It is difficult to give general advice on the type of body fluid that is most suited to perform IMD diagnostics. Urine and blood are easily accessible, with urine often serving as the first diagnostic approach. The kidneys rapidly excrete many metabolites, while others preferentially remain in the bloodstream. Both urine‐ and plasma‐based MS metabolomics are used for the diagnosis of metabolic disorders (urine: [35]; plasma: [36, 37]). Additionally, MS‐based metabolomics of cerebrospinal fluid (CSF) may provide valuable insights for patients suspected of having neurometabolic disorders [38, 39]. Blood cells as a sample source may provide advantages to diagnose additional IMDs due to a different coverage of the (intracellular) metabolome. Until now, standardized metabolomics analysis for IMDs in blood cells has not been widely reported.

### 2.2 Sample Measurement and Detection of Metabolites

Various analytical techniques have been developed to measure metabolites, including liquid and gas chromatography, nuclear magnetic resonance spectroscopy (NMR), and MS. In recent years, the growing demand for highly selective and sensitive methods for reliable metabolite detection has led to the increasing popularity of MS coupled with liquid chromatography (LC–MS) in metabolomics. While sample preparation can be optimized to target specific metabolite classes (e.g., polar vs. apolar metabolites), the process is generally straightforward, primarily involving metabolite extraction. Liquid chromatography is used to separate isomeric metabolites—compounds with the same molecular formula and *m/z* but different structures—thereby enhancing the sensitivity of MS. This separation is achieved based on a metabolite's affinity to the chromatographic column, which is typically determined by a specific chemical property, such as polarity. However, the choice of chromatographic method (e.g., reversed‐phase, hydrophilic interaction liquid chromatography [HILIC], etc.) and corresponding sample preparation can introduce biases, focusing the analysis on certain chemical categories of the metabolome (e.g., lipids vs. water‐soluble metabolites). MS can be performed in both positive and negative ionization modes, generating cationic (protonated or metal ion adducts) or anionic (primarily deprotonated) forms of metabolites, respectively. To maximize metabolome coverage and obtain complementary data for metabolite identification, conducting LC–MS experiments in both ionization modes is recommended whenever feasible. With recent advances in MS technology, untargeted metabolomics has become increasingly sensitive and comprehensive, enabling the detection of thousands of unique metabolites in a single experiment [40, 41, 42]. Although untargeted methods typically do not aim towards absolute quantitation (at least not for most of the detected features), they allow for relative quantitation, enabling comparisons between different sample groups (e.g., patients vs. controls) or longitudinal comparisons for the same patient. These methods also enable the interpretation of data from a single patient sample by comparing it with the data from control samples measured in the same run. Despite the goal of untargeted metabolomics to detect all metabolites, there is no single chromatographic method that offers unbiased coverage of the entire metabolome. Thus, selecting an appropriate chromatographic technique based on the analytes to be measured remains a critical factor in experimental design. Overall, the broad metabolome coverage provided by untargeted metabolomics allows us to distinguish between metabolic disorders with multiple subtypes, for example, hyperprolinemia type I and type II [43]. In the case of hyperprolinemia, distinguishing subtypes requires the detection of metabolites from different classes. Hyperprolinemia type I results from a deficiency in proline dehydrogenase, leading to an isolated accumulation of proline. In contrast, hyperprolinemia type II is caused by a defect in Δ^1^‐pyrroline‐5‐carboxylate dehydrogenase, resulting in the accumulation of not only proline but also additional metabolites, including pyrrole‐2‐carboxylic acid, 2‐pyrroloyl‐glycine, and pyrroline‐5‐carboxylate (P5C) derivatives. Untargeted metabolomics enables precise subtype differentiation by detecting these distinct metabolic signatures, emphasizing the necessity of multi‐class metabolite profiling in the classification and diagnosis of metabolic disorders.

### 2.3 Data Preprocessing

A single untargeted MS‐based metabolomics run measuring multiple samples generates an enormous amount of data, typically about 20 000 metabolic features detected per sample. During sample measurement, multiple technical challenges including shifts in retention times during longer consecutive measurements, batch effects, and the possible presence of various artifacts, can potentially hinder data interpretation. Hence, it is crucial to preprocess untargeted metabolomics datasets to extract and interpret meaningful biological insights [44, 45, 46, 47]. General data preprocessing steps include peak detection, alignment, and normalization. Various open‐source computational tools like XCMS [48], MZmine [49], etc. can be used to perform data preprocessing, apart from conventional instrument vendor software. It is also important to consider that the mass features detected are not always metabolites but related species such as isotopes, adducts, and in‐source fragments of a single metabolite with different *m/z* values [50]. To tackle this issue, computational tools such as CAMERA [51] and xMSannotator [52] can be used to help identify and cluster mass features that likely belong to the same metabolite. This feature matrix is then used to perform further downstream data analysis depending on the research question. The output of data preprocessing is a high‐dimensional feature matrix where variables are the detected mass features (with their corresponding m/z and RT values) associated with an intensity value (or area under the peak), indicating its relative abundance in a specific sample.

### 2.4 Metabolite Annotation

Feature annotation is the process of transforming detected molecular features into identified metabolites, ultimately providing biologically meaningful insights. In LC–MS‐based untargeted metabolomics, a standard approach for feature annotation involves matching experimental accurate mass (*m/z*) values with those from in‐house standards or public databases using a user‐defined mass tolerance window to generate a list of possible candidate metabolites in the latter scenario. The identification of mass features in untargeted metabolomics experiments is categorized into five confidence levels, as defined by the Metabolomics Standards Initiative [53] and further refined with advancements in high‐resolution MS [54]. The accuracy and robustness of the identification depend on whether single or multiple data types are applied and whether experimental data from biological samples are compared with those from authentic chemical standards. At *level 5* of metabolite annotation, a feature is assigned based on its mass (*m/z*), without information about its chemical formula or structure. *Level 4* annotation allows the determination of a chemical formula using accurate mass spectral data, and adducts, isotopes, or fragment information. The formula can then be matched to databases such as the HMDB, ChemSpider, or METLIN, though this level does not provide sufficient evidence to propose a specific structure. *Level 3* corresponds to tentative identification, where potential structures can be suggested, but there is not enough data to confirm a single structure. At *level 2*, an exact structure can be proposed based on tandem MS data, retention time (RT), and comparison with reference standards in databases. The highest confidence level, *level 1*, confirms the structure of a metabolite through comparison with a reference standard, supported by multiple orthogonal techniques such as RT, *m/z*, MS^n^, collision cross‐section measurements, and/or infrared ion spectroscopy (IRIS).

### 2.5 Data Analysis and Interpretation

Depending on the biological research question, analysis and interpretation of untargeted metabolomics data usually involve the application of univariate and multivariate statistical approaches to prioritize mass features that are significantly altered among the experimental groups (e.g., healthy vs. diseased) and reveal variation patterns that may correlate with the phenotype. The altered mass features derived after statistical analysis of MS1 measurements can further be accessed based on their biochemical relevance by performing pathway enrichment analysis, also called metabolite set enrichment analysis [55, 56, 57]. Computational tools like mummichog [58] and PIUMet [59] help in doing this by prioritizing mass features with candidate matches that can be mapped onto a metabolic network derived from available pathway databases to predict the pathway activity. This approach can also be used to resolve the identity of the unknown metabolic features by selectively targeting them for MS^2^ analysis.

In recent years, multiple metabolic diagnostic laboratories have implemented (untargeted and semi‐targeted) MS‐based metabolomics workflows as a screening tool for patients suspected of having an IMD. Examples include ultra‐high‐performance pentafluorophenylpropyl phase‐based ultra‐high performance‐liquid chromatography (UHPLC)‐Orbitrap MS‐based untargeted metabolomics workflow [36], direct infusion Q‐Exactive MS‐based untargeted metabolomics workflow for plasma and dried blood spots [60], the targeted urine ultra‐high‐performance liquid chromatography‐quadrupole time‐of‐flight MS (UHPLC‐qTOF‐MS) based metabolomics workflow [35] and several other labs and commercial providers [29, 31, 61, 62, 63, 64, 65, 66].

**FIGURE 1:**
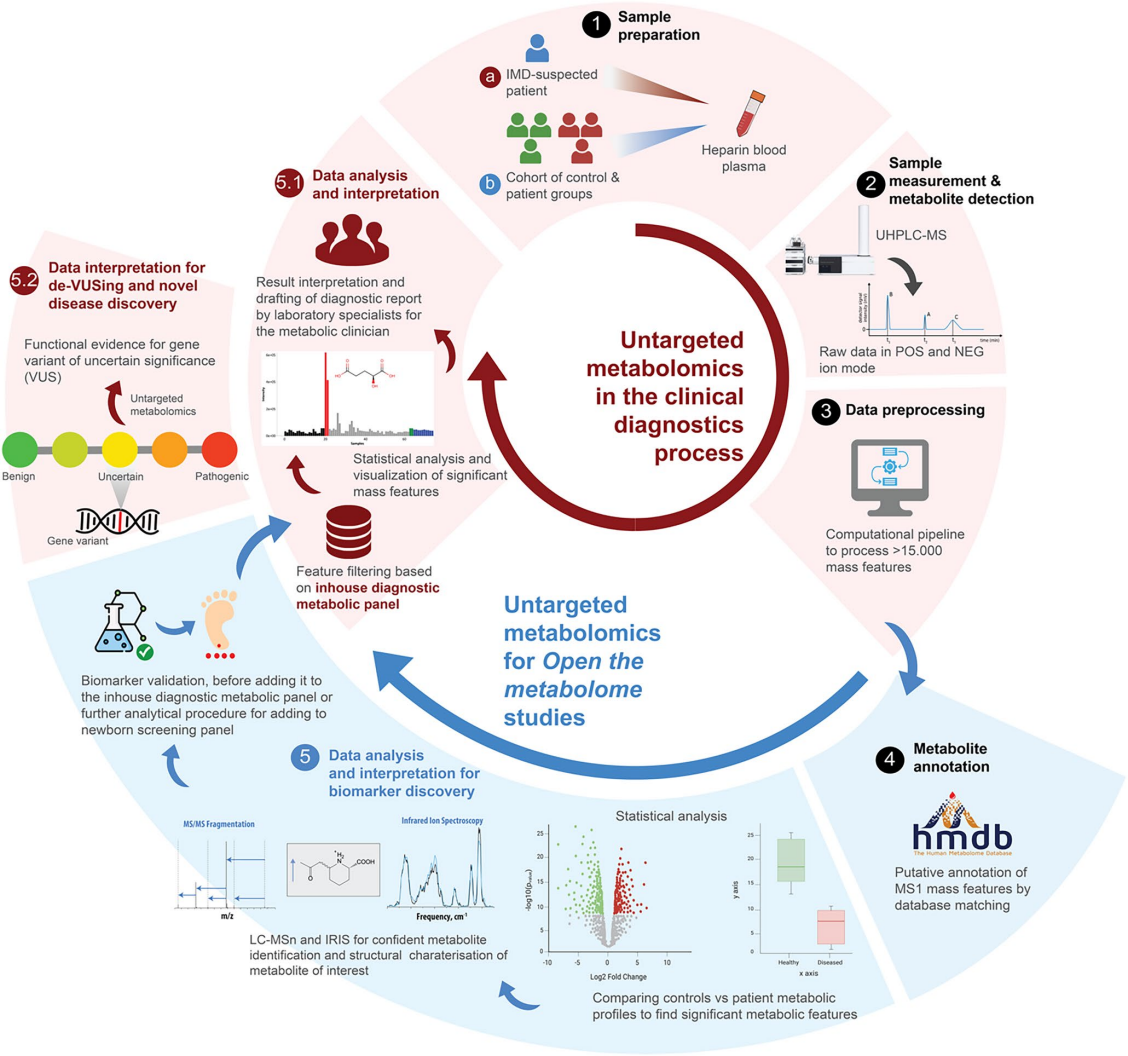
MS‐based untargeted metabolomics workflow implemented for use in the clinical diagnostic process (steps 1a to 5.1, in *red*), *open metabolome* studies (steps 4 and 5, in *blue*), and de‐VUSing (step 5.2, in *red*). *Red* blocks represent the standard diagnostic workflow followed for patients suspected of having an IMD. This process only looks at biomarkers relevant for a broad array of metabolic disorders, based on the in‐house diagnostic metabolic panel. *Blue* blocks illustrate the *open the metabolome* approach, a research method for comparing cohorts of patients and controls (step 1b), or even for single‐patient metabolome exploration. Steps 1–4 are shared between diagnostic and research workflows, differing mainly in the sample grouping: Individual patient analysis for diagnostics (1a) versus cohort‐based comparison for *open the metabolome* studies (1b). De‐VUSing (step 5.2, in *red*) involves data interpretation at the individual patient profile level to aid in variant classification.

An untargeted metabolomics‐based approach has been implemented in our laboratory based on reversed‐ phase UHPLC‐qTOF‐MS method [37] supplemented with a computational pipeline [67, 68]. This metabolomics workflow is representative of methods commonly used in diagnostic and research laboratories and therefore we describe it in detail. The complete workflow is compatible with ISO:15189 accredited clinical diagnostics and since July 2020 has notably been used as a first‐tier test for diagnostic screening of IEMs. As a part of this workflow, patient‐derived plasma samples are measured in an untargeted manner using reverse‐phase high‐performance LC/MS (Figure 1 (Steps 1a and 2)). The acquired data is pre‐processed using an in‐house computational pipeline to generate a feature matrix (Figure 1 (Step 3)). The MS1 feature data is matched against the Human Metabolome database (HMDB) [69] to perform putative metabolite annotation (Figure 1 (Step 4)). The feature matrix is then used for further analysis and interpretation. For application in IMD diagnostics, the feature matrix is filtered based on an in‐house diagnostic metabolic panel and a list of pre‐set filtering parameters to reduce the feature space and interpret the detected mass features in a targeted manner (Figure 1 (Step 5)). This metabolic panel contains 87 IMDs and their associated biochemical markers (refer to File S1 of the supplementary section for the complete list) [33, 37].

## 3 Open the Metabolome: Applications of Untargeted Metabolomics in Clinical Research

For IMD diagnostics, only a subset of the measured features is reported to the referring clinician based on the metabolic panel. Nevertheless, the entire recorded metabolome remains available to perform “Open the metabolome” analysis [37], a term coined to represent the exploratory analysis of untargeted metabolomics data. This has relevance, not only for the discovery of additional potential diagnostic and prognostic biomarkers for the expansion of the metabolic panel but also in understanding disease pathophysiology (Figure 1 (Steps 1b‐5)). The application of an *Open the metabolome* approach to 21 IMDs led to the identification of novel biomarkers in majority of these diseases (19 of 21 IMDs of which 3 or more patients were analyzed = 90%), illustrating the promise for novel diagnostic biomarkers and novel insights in disease mechanisms. Below, we describe five application areas of untargeted metabolomics in clinical translational research: (a) identification of novel biomarkers and disease insights, (b) unraveling disease pathophysiology, (c) monitoring disease progression and therapy response, and (d) Interpretation of gene variants for the characterization of IMDs.

### 3.1 Identification of Novel Biomarkers and Disease Insights

#### 3.1.1 Pyridoxine‐Dependent Epilepsy (PDE)

**FIGURE 2:**
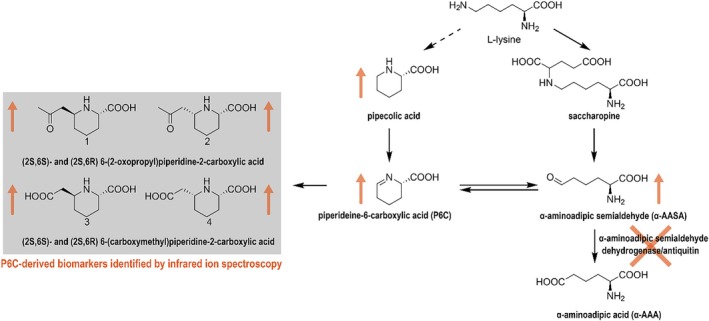
Overview of L‐lysine pathway affected in pyridoxine‐dependent epilepsy (PDE) Inactivity of the enzyme antiquitin (ALDH7A1 gene) causes accumulation of α‐aminoadipic semialdehyde (α‐AASA) and piperideine‐6‐carboxylic acid (P6C). P6C can react with β‐ketoacids or β‐diacids, forming P6C‐derived biomarkers ((2S,6S)/(2S,6R)‐6‐(2‐oxopropyl)piperidine‐2‐carboxylic acid (2‐OPP) and (2S,6S)/(2S,6R)‐6‐(carboxymethyl)piperidine‐2‐carboxylic acid (2‐CMP)) [70].

Pyridoxine‐dependent epilepsy (PDE‐ALDH7A1, OMIM 266100) is an IMD affecting lysine metabolism, leading to refractory seizures in newborns. As illustrated in Figure 2, the condition is caused by a deficiency of α‐aminoadipic semialdehyde dehydrogenase (antiquitin), resulting in the accumulation of alpha‐aminoadipic semialdehyde (α‐AASA) and piperidine‐6‐carboxylate (P6C). These metabolites chemically inactivate pyridoxal 5′‐phosphate (PLP), the active form of vitamin B6, leading to PLP deficiency. Treatment typically involves high‐dose vitamin B6 supplementation, often combined with lysine restriction and arginine supplementation, which generally controls seizures. However, intellectual disability remains a common challenge. Early diagnosis through newborn screening could improve outcomes, but the lack of reliable biomarkers has hindered progress. The measurement of α‐AASA and P6C is unsuitable for dried blood spot screening due to their chemical instability. Given that PDE‐ALDH7A1 is both treatable and relatively common (estimated incidence of 1:65000 to 1:250000 live births [71]), untargeted metabolomics was employed to identify new biomarkers for neonatal screening. In 2021, using LC–MS‐IRIS, (2S,6S)/(2S,6R)‐6‐(2‐oxopropyl)piperidine‐2‐carboxylic acid (2‐OPP) and (2S,6S)/(2S,6R)‐6‐(carboxymethyl)piperidine‐2‐carboxylic acid (2‐CMP) were identified as novel plasma biomarkers for PDE‐ALDH7A1 [72]. Among these, 2‐OPP has since been shown to be suitable for dried blood spot‐based screening protocols. Furthermore, a stable isotope internal standard based on 2‐OPP is now routinely used in our metabolomics laboratory for quantitative measurements in diagnostics and for patient monitoring.

The discovery of novel metabolites not only opens new avenues for diagnostic biomarkers but also provides deeper insights into pathophysiology and the underlying biochemical mechanisms. In zebrafish larvae, 2‐OPP was found to induce hyperactivity resembling epilepsy, indicating that its levels should be minimized in patients with PDE. By examining the chemical structure of 2‐OPP, we elucidated its non‐enzymatic formation as a reaction between P6C and acetoacetic acid (AcAc). This insight led to a novel clinical recommendation to avoid ketosis, a state in which AcAc levels are elevated, in PDE patients [72].

#### 3.1.2 Hyperprolinemia Type II (HPII)

**FIGURE 3:**
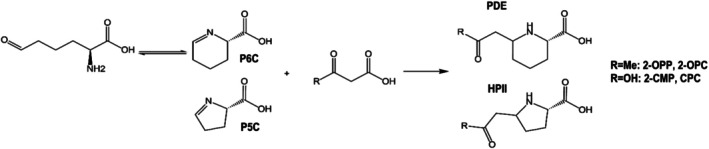
Spontaneous chemical reactions lead to the formation of biomarkers. Uncovering the underlying chemistry leads to generalized observations that can predict the presence and structures of relevant metabolites involved in analogous chemistry. P6C and P5C have similar chemical structures and analogous reactivity given closely related disease‐specific metabolites. These structures are shown on the right side of the figure where “R” can be a methyl group (giving 2‐OPP in PDE and 2‐OPCin HPII) or a hydroxyl group (giving 2‐CMP in PDE and CPC in HPII).

Additionally, uncovering the previously unknown mechanisms of formation of 2‐OPP and 2‐CMP as adducts of P6C with AcAc or malonic acid in PDE provided new biochemical insights relevant to other inborn errors of metabolism. Recognizing that P6C can react as an imine with any carbonyl‐containing species that have stable enol tautomers led us to hypothesize that its five‐membered ring analog, P5C, would likely form similar metabolites, illustrated in Figure 3. This led to the discovery of (2 R)‐5‐(2‐oxopropyl)pyrrolidine‐2‐carboxylic acid (2‐OPC) and (2 R)‐5‐(carboxymethyl)pyrrolidine‐2‐carboxylic acid (CPC) as novel biomarkers for hyperprolinemia type II (HPII), a disease in which P5C accumulates [43]. These novel biomarkers were shown to distinguish between Hyperprolinemia types I and II.

### 3.2 Unraveling Disease Pathophysiology

#### 3.2.1 Glucose Transporter Type‐1 Deficiency Syndrome (GLUT1DS)

Using untargeted metabolomics and IRIS, novel biomarkers for glucose transporter type 1 deficiency syndrome (GLUT1DS) were identified in CSF [73]. Notably, xylose‐α1‐3‐glucose and xylose‐α1‐3‐xylose‐α1‐3‐glucose oligosaccharides were discovered, the first time that these molecules were detected in non‐conjugated form in body fluids though they are known as covalently bound O‐glycans in several proteins [74, 75]. Both compounds were found at reduced concentrations in CSF samples from GLUT1DS patients, including those on a ketogenic diet. These O‐glycans are thought to derive from glycosylated proteins, and their diminished levels in GLUT1DS are likely due to insufficient glucose, a key substrate for the synthesis of UDP‐glucose as a precursor for this type of O‐glycan. This form of O‐glycosylation may play a role in cellular processes that contribute to the pathophysiology of GLUT1DS. The findings suggest that glucose deficiency in the brains of GLUT1DS patients may affect more than just energy metabolism. Since the ketogenic diet provides ketones as an alternative energy source but not glucose, it is unlikely to normalize this specific O‐glycosylation pathway. Therefore, a ketogenic diet alone may be insufficient to fully restore O‐glycan synthesis meaning that further research is needed to determine the optimal therapeutic approach for GLUT1DS.

### 3.3 Monitoring Disease Progression and Therapy Response

#### 3.3.1 Phenylketonuria (PKU)

Traditionally, management of PKU has largely relied on monitoring blood phenylalanine (Phe) concentrations. While there is a well‐documented general correlation between Phe levels and cognitive and behavioral outcomes, a subset of poor outcomes remains unexplained despite careful Phe control. This has prompted researchers to explore broader metabolic disturbances in these patients to identify potential for improvements in treatment.

Several untargeted metabolomics studies into PKU demonstrated the potential for uncovering novel biomarkers that can significantly enhance our understanding of both disease pathophysiology and treatment strategies. Václavík et al. employed untargeted metabolomics and multistage fragmentation MS to elucidate novel compounds in the plasma of PKU patients [76]. Among these compounds were two new conjugates: glutamyl‐glutamyl‐phenylalanine and phenylalanine‐hexose. Interestingly, these markers exhibited high inter‐patient variability and did not closely correlate with Phe levels, suggesting that they may be independently informative for patient monitoring.

Building upon earlier findings, in‐depth metabolomics investigations by van der Weerd et al. and van Wegberg et al. have identified complex metabolic alterations that extend well beyond Phe accumulation and correlate more closely with the clinical status of PKU patients [77, 78]. A distinct metabolic fingerprint of PKU emerged, characterized by elevated levels of several Phe catabolites and conjugates in the blood, including the Phe‐hexose conjugate previously observed by Václavík et al. Among these, N‐lactoyl‐Phe was shown to be particularly useful as a prognostic biomarker, demonstrating a stronger association with working memory and mental health outcomes than Phe measurements alone. Additional metabolic disruptions in PKU were shown to involve elevated tryptophan derivatives, specifically increased concentrations of certain indole and pyridine compounds, which may be linked to deviations in the gut–brain axis, indicating another potential influence on PKU pathophysiology.

Van Outersterp et al. used IRIS to show that the Phe‐hexose metabolite observed previously was specifically Phe‐glucose and formed as an Amadori rearrangement product [79]. These products, previously well‐known as intermediates in the formation of advanced glycation end‐products, highlight broader metabolic disruptions that could play a role in the pathophysiology in PKU as well as other metabolic disorders that lead to elevated levels of amino acids in the body where analogous Amadori products can also spontaneously form.

#### 3.3.2 Late Infantile Neuronal Ceroid Lipofuscinosis (CLN2)

Untargeted metabolomics has also been used for monitoring disease progression and therapy response in other disorders such as late infantile neuronal ceroid lipofuscinosis (CLN2 disease) [80]. Novel CLN2 biomarkers in CSF were identified including several acetylated species that were present at decreased levels. Of more than 1400 metabolites profiled, 29 were found to linearly correlate with disease severity scores. Furthermore, the authors of that study hypothesized that since acetate is necessary for mitochondrial respiration, a deficiency of acetylated species in CSF implies that an energy deficit may be a driver of neurodegeneration in CLN2.

#### 3.3.3 Mitochondrial Encephalomyopathy, Lactic Acidosis, and Stroke‐Like Episodes (MELAS)

Another example comes from mitochondrial diseases, such as mitochondrial encephalomyopathy, lactic acidosis, and stroke‐like episodes (MELAS), which are caused by the m.3243A > G mutation. This condition is difficult to diagnose because of its high phenotypic variability and unpredictable severity. Sharma et al. showed that untargeted metabolomics can be used to analyze plasma samples from highly phenotyped patients and identify metabolites that reliably distinguish MELAS patients from controls [81]. Furthermore, they uncovered novel markers, such as N‐lactoyl‐amino acids, β‐hydroxy acylcarnitines, and β‐hydroxy fatty acids, which correlate strongly with disease severity (Karnofsky performance).

#### 3.3.4 Empagliflozin Dose Responsiveness in Glycogen Storage Disorder Type Ib (GSD‐Ib)

Finally, untargeted metabolomics can be used to assess the biochemical result of therapy and disease progression [82, 83, 84, 85]. In a 2022 study, empagliflozin, a kidney sodium‐glucose co‐transporter‐2 (SGLT2) inhibitor, was shown to reduce plasma 1,5‐anhydroglucitol levels as well as toxic derivatives in neutrophils in a GSD‐Ib patient using untargeted metabolomics. This enabled the assessment of dosage response and dietary management [86].

### 3.4 Interpretation of Gene Variants for the Characterization of IMDs

When conducting WES or WGS on a patient with a disorder of unknown etiology, approximately 50%–60% will remain without a diagnosis and VUS are often encountered [87]. A VUS is a genetic variant that cannot be definitively classified [88]. Variant interpretation and classification follow the American College of Medical Genetics and Genomics (ACMG) and the Association for Molecular Pathology (AMP) guidelines [89] into five‐tier classes: pathogenic, likely pathogenic, uncertain significance, likely benign, and benign. Often, these variants are so rare in the population that there is limited information available. Typically, additional information is required to determine whether the variant is associated with a disease. In such cases, functional validation is performed to obtain conclusive evidence for the pathogenicity of the variants. Different approaches have been defined to perform functional validation studies of genetic variants [90]. However, customizing assays for each new variant encountered in WES/WGS data can be a time‐consuming process. Since untargeted metabolomics offers an unbiased profile of all metabolites present, including significantly altered ones, it can pinpoint defective pathways and pathogenic biochemical processes. This can allow for a targeted evaluation of the associated genes in genomic data. Additionally, (un)targeted metabolomics, when used for *de‐VUSing* (a process of going from a VUS to functional/biochemical evidence), can aid in interpreting potential deleterious functional effects of these variants and their overall pathogenicity and uncover novel biomarkers for known diseases [91, 92].

#### 3.4.1 TMLHE‐Linked Autism

In a recent example of gene‐variant interpretation with untargeted metabolomics, a patient with autism spectrum disorder was found to carry a novel variant in the *TMLHE* gene (MIM: 300777) [93]. Metabolomic profiling revealed increased trimethyllysine (TML) and decreased γ‐butyrobetaine (γ‐BB) levels. The *TMLHE* gene is involved in the pathway for the synthesis of L‐carnitine in which TML and γ‐BB are intermediates. This distinct metabolic disruption provides functional evidence for the pathogenicity of this gene and provides the basis for a disruption of L‐carnitine biosynthesis. In clinical practice globally, the initial analysis of whole‐exome sequencing (WES) typically relies on gene panels composed of genes already linked to known diseases (though we note that broader analysis is increasingly being applied in rare diseases). In this context, de‐VUSing through targeted metabolomics—by examining both the substrate and product of the implicated enzyme, as illustrated in the *TMLHE* gene example above—is a valuable strategy. However, because this method focuses on established disease‐associated genes, its capacity to uncover novel IMDs is limited.

#### 3.4.2 N‐Acetylneuraminate Synthase (NANS) Deficiency

An example of gene‐variant interpretation supported by untargeted metabolomics to discover a novel IMD is presented in a 2016 study describing the N‐Acetylneuraminate Synthase (NANS) deficiency [94]. Prior to this study, the *NANS* gene had not been associated with any disease and was absent from existing gene panels. The investigation began with whole‐exome sequencing (WES) of a patient exhibiting intellectual developmental disorders and skeletal dysplasia, which identified a VUS. Untargeted metabolic screening of CSF and plasma of this patient detected the accumulation of N‐acetyl‐D‐mannosamine (ManNAc). This biochemical finding directed the interpretation of the WES data and led to the conclusion that mutations in the *NANS* gene, encoding an enzyme in the CMP‐sialic acid biosynthesis, were pathogenic and causative for the clinical features in this patient. This example highlights the crucial role of the open metabolome approach in uncovering novel IMDs.

## 4 Challenges of Untargeted Metabolomics in the Context of IMDs

As described in the previous sections of this review, LC/MS‐based untargeted metabolomics is a mature technology that has the potential to provide an expansive and unbiased profile of the metabolome, and it is currently proliferating as a powerful methodology for the diagnosis and study of IMDs in clinical laboratories. However, despite this potential, several significant challenges remain, particularly in metabolite identification and in its application to rare diseases such as IMDs. In this section, we will give an overview of several of these technical, biological, and practical challenges.

Perhaps the primary bottleneck in untargeted metabolomics remains the identification of (unknown) metabolites. The human metabolome includes an extraordinarily diverse set of thousands of molecules with a wide range of characteristics, including but not limited to molecular weight, polarity, stability, volatility, and chemical functionality. Moreover, existing databases of metabolites and biomarkers, including the HMDB [69] and MarkerDB [95], remain incomplete in terms of the absence of unknown metabolites and data coverage for known or expected metabolites. This means that a significant proportion of detected mass features in untargeted metabolomics data remain unannotated or only tentatively identified [53]. This part of the metabolome has been referred to as the “dark matter of the metabolome”. The challenge of shedding light on this dark matter is made worse by the enormous complexity of isomeric structures and the lack of universally applicable identification tools [50] for molecular structure elucidation. Especially for rare diseases, where novel metabolites can often play a crucial diagnostic role as biomarkers or have pathophysiological significance, the difficulty in accurately identifying metabolites remains a major bottleneck. Techniques such as tandem mass spectrometry (MS^n^), ion mobility spectrometry (IMS) and NMR spectroscopy have each been explored as tools for molecular structure identification of metabolites leading to *level 1* metabolite identification. However, none of these techniques provide general solutions and typically only provide partial insights (tandem MS fragmentation patterns and collisional cross sections) or are severely limited in terms of their sensitivity (NMR). Furthermore, MS^n^ and IMS heavily rely on the availability of chemical standards, which strongly limit their utility for novel compounds [46]. On the other hand, NMR can often provide definitive structural assignments; however, its limited sensitivity (micromolar range) makes it impractical in most cases. Very recently, emerging technologies, based on IRIS, have shown promise in overcoming these limitations. Currently, these techniques remain limited to only a few specialized laboratories worldwide; however, we expect this technology to more widely proliferate in the coming years [79, 96].

Another challenge relates to the high‐dimensional data generated by untargeted metabolomics measurements. Several factors make this data difficult to analyze. Firstly, the number of detected features is much larger than the number of samples, a problem often termed as ‘Curse of dimensionality’. Due to this, many significance‐based statistical approaches are highly vulnerable to the problem of overfitting. Moreover, a detected metabolic feature does not necessarily directly translate into a metabolite. This is because the LC–MS technique is highly redundant due to the recurrent detection of numerous peaks from isotopes, adducts, in‐source fragments, and contaminants, which greatly increase the total number of features detected and make data interpretation further complicated. Some of the measured features can also arise from medication and diet. Both these factors can significantly alter metabolic profiles, making it challenging to distinguish between changes due to external influences and those inherent to the biological condition being studied.

As rare diseases, individual IMDs are mostly present in very limited patient populations. This presents inherent challenges in setting up metabolomics studies with sufficient statistical power. Limited sample groups make distinguishing meaningful metabolic variations from noise more difficult [59]. Furthermore, inter‐patient metabolic variability which is influenced by external factors such as age, gender, BMI, diet, comorbidities, lifestyle and environment adds to the issue of limited patient group size. Finally, varied sample collection protocols, storage conditions, and time from collection also add complexity to data interpretation [16]. Recently, innovative strategies integrating multi‐omics datasets and methods using machine learning are highly valuable for generating statistically meaningful insights from limited data [17].

Another challenge related to the rarity of IMDs is that they often involve poorly or partially characterized metabolic pathways. Unlike targeted metabolomics, where the focus is on predefined metabolites as biomarkers, untargeted metabolomics aims to uncover novel metabolic perturbations where the biological relevance is often unknown [97]. Translating these metabolic observations into clinically relevant insights is challenging and often involves consideration of other interconnected metabolic pathways and related genetic findings. Additionally, correlating metabolomics data to patient phenotypes is as well naturally a challenge when working with a limited cohort size. This relates to difficulties establishing validated reference ranges of metabolite levels, especially for novel metabolites where internal standards required for quantitative measurements are unavailable [30]. Pathway analysis of metabolites creates another challenge in untargeted metabolomics data interpretation. These approaches were initially developed for gene expression data and are often misapplied in metabolomics and lead to misleading biological interpretations since metabolite levels lack the coherence and localization seen in gene expression. Bioinformatic and analytical limitations further complicate accurate metabolomics‐based pathway analysis. Hence, cautious use, better validation, and deeper biological collaboration to improve interpretation accuracy is recommended [98].

In summary, enhancing the completeness of metabolite databases, developing robust analytical and computational technologies for identification and structure elucidation, and creating sophisticated data interpretation tools are all areas that currently appear likely to overcome many of these challenges in the upcoming years. Thus, we envision that untargeted metabolomics will continue to expand as a tool in metabolic laboratories for discovering novel biomarkers, understanding pathophysiological mechanisms, and improving therapeutic outcomes in patients with IMDs and other rare diseases.

Major challenges of applying untargeted metabolomics in the field of IMDs

- **Annotation ambiguity:** A large proportion of detected features remain unidentified due to complexity of the generated data and limitations in spectral databases and standards. Additionally, isomers and structurally similar compounds are often indistinguishable using standard MS‐based techniques.
- **Curse of dimensionality and limited cohort size:** Untargeted metabolomics generates high‐dimensional data with thousands of variables (metabolic features) but often involves very limited cohort size, due to the inherent rarity of IMDs. This hampers statistical power, increases the risk of overfitting, and complicates biomarker discovery.
- **Partially characterized metabolic pathways:** Many metabolic pathways involved in inherited metabolic disorders remain partially characterized. This limits the interpretation of untargeted metabolomics data and the identification of novel disease‐related biomarkers.

## 5 Outlook

Untargeted metabolomics has significantly enhanced our mechanistic understanding of the complex and diverse pathogenesis and pathophysiology of IMDs. An ever‐expanding number of research groups and metabolic diagnostic laboratories are implementing it for patient diagnostics, biomarker discovery, and personalized medicine. Furthermore, novel metabolic insights are increasingly aiding in the identification of novel therapeutic targets and enhancing disease and treatment‐monitoring abilities.

While traditional targeted assays currently remain important for quantifying known biomarkers, untargeted metabolomics provides a unique and unbiased assessment of broader portions of the metabolome. This enables the discovery of novel biomarkers and deepens our understanding of disease mechanisms, especially crucial for rare and less‐studied IMDs. Another significant advantage of applying untargeted metabolomics lies in its ability to complement genetic diagnostics (WES/WGS) by functionally clarifying variants of unknown significance (de‐VUSing), providing often critical biochemical evidence to confirm or refute the pathogenicity of genetic variants. Further exploration of the intracellular metabolome and the use of advanced model systems (e.g., organoids, induced pluripotent stem cells, animal models) will expand our understanding of metabolic disorders at the organ and cellular level. This expanded scope of metabolomics promises to offer a whole new range of opportunities for diagnostic, prognostic and treatment‐monitoring biomarkers.

Another emerging application area of untargeted metabolomics is for standardized clinical phenotyping based on the broad biochemical profile of patients in the absence of an existing diagnosis. Characterizing disruptions in metabolic profiles relative to a control population provides disease‐specific fingerprints, groups patients based on metabolic disruptions and thus supports clinical diagnostics even in challenging cases.

Future advances in untargeted metabolomics are expected to address existing challenges such as incomplete metabolite databases, analytical limitations, and the handling of complex and high‐dimensional datasets. Innovations in computational biology, bioinformatics, and the continued integration of multi‐omics datasets using artificial intelligence and machine learning techniques will surely play pivotal roles in overcoming these challenges. Advanced and emerging analytical technologies, including IRIS, are currently being incorporated into metabolomics workflows to overcome limitations in metabolite identification. Using MS imaging, spatially resolved (un)targeted metabolomics is an emerging approach that offers the potential to connect metabolic disruption to tissue‐specific and spatially resolved patho‐mechanisms. Overall, untargeted metabolomics is poised to become an integral tool in precision medicine, significantly advancing our understanding and treatment of inherited metabolic disorders.

Key insights and benefits of applying untargeted metabolomics in the clinical laboratory

- Discovery of (novel) diagnostic, prognostic, and monitoring biomarkers.
- Functional validation of genetic variants (de‐VUSing), providing biochemical evidence for pathogenicity.
- Deepened insights into disease pathophysiology, uncovering underlying biochemical mechanisms supporting the discovery of novel IMDs.
- Enhanced patient stratification and precision medicine through comprehensive metabolic profiling.
- Complementary integration with targeted metabolomics and genomics techniques for holistic patient diagnostics.

## Author Contributions

J.M. and P.K. wrote the manuscript. U.F.H.E. compiled the summarized IMD biomarker panel. All authors conceptualized, critically reviewed, and edited the manuscript and agree to submit for publication the final version.

## Funding

This work was supported by Nederlandse Organisatie voor Wetenschappelijk Onderzoek.

## Conflicts of Interest

The authors declare no conflicts of interest.

## Supporting information


**Data S1:** In‐house list of IMDs and their associated biomarkers.

## Data Availability

The data that supports the findings of this study is available in the Supporting Information of this article.
